# An elevated expression of serum exosomal microRNA-191, − 21, −451a of pancreatic neoplasm is considered to be efficient diagnostic marker

**DOI:** 10.1186/s12885-018-4006-5

**Published:** 2018-01-31

**Authors:** Takuma Goto, Mikihiro Fujiya, Hiroaki Konishi, Junpei Sasajima, Shugo Fujibayashi, Akihiro Hayashi, Tatsuya Utsumi, Hiroki Sato, Takuya Iwama, Masami Ijiri, Aki Sakatani, Kazuyuki Tanaka, Yoshiki Nomura, Nobuhiro Ueno, Shin Kashima, Kentaro Moriichi, Yusuke Mizukami, Yutaka Kohgo, Toshikatsu Okumura

**Affiliations:** 1 Division of Gastroenterology and Hematology/Oncology, Department of Medicine, Asahikawa Medical University, 2-1 Midorigaoka-higashi, Asahikawa, Hokkaido 078-8510 Japan; 2 Department of Gastroenterology, International University of Health and Welfare Hospital, Nasushiobara, Japan

**Keywords:** Pancreatic cancer, Exosome, microRNA-21, microRNA-451a, Intraductal papillary mucinous neoplasm, Tumor marker

## Abstract

**Background:**

Pancreatic cancer is associated with an extremely poor prognosis, so new biomarkers that can detect the initial stages are urgently needed. The significance of serum microRNA (miR) levels in pancreatic neoplasm such as pancreatic cancer and intraductal papillary mucinous neoplasm (IPMN) diagnosis remains unclear. We herein evaluated the usefulness of miRs enclosed in serum exosomes (ExmiRs) as diagnostic markers.

**Methods:**

The ExmiRs from patients with pancreatic cancer (*n* = 32) or IPMN (*n* = 29), and patients without neoplasms (controls; *n* = 22) were enriched using ExoQuick-TC™. The expression of ExmiRs was evaluated using a next-generation sequencing analysis, and the selected three miRs through this analysis were confirmed by a quantitative real-time polymerase chain reaction.

**Results:**

The expression of ExmiR-191, ExmiR-21 and ExmiR-451a was significantly up-regulated in patients with pancreatic cancer and IPMN compared to the controls (*p* < 0.05). A receiver operating characteristic curve analysis showed that the area under the curve and the diagnostic accuracy of ExmiRs were 5–20% superior to those of three serum bulky circulating miRs (e.g.; ExmiR-21: AUC 0.826, accuracy 80.8%. Circulating miR-21: AUC 0.653, accuracy 62.3%). In addition, high ExmiR-451a was associated with mural nodules in IPMN (*p* = 0.010), and high ExmiR-21 was identified as a candidate prognostic factor for the overall survival (*p* = 0.011, HR 4.071, median OS of high-ExmiR-21: 344 days, median OS of low-ExmiR-21: 846 days) and chemo-resistant markers (*p* = 0.022).

**Conclusions:**

The level of three ExmiRs can thus serve as early diagnostic and progression markers of pancreatic cancer and IPMN, and considered more useful markers than the circulating miRs (limited to these three miRs).

**Electronic supplementary material:**

The online version of this article (10.1186/s12885-018-4006-5) contains supplementary material, which is available to authorized users.

## Background

Pancreatic cancer (PC) is a frequent cause of cancer death worldwide [1]. While advances in clinical treatments, including chemotherapy and surgery, have improved the prognosis of PC in the past decades, the early detection of PC remains quite difficult. Thus, the prognosis of PC remains poor, even when using advanced imaging techniques such as computed tomography or positron emission tomography. Carbohydrate antigen 19–9 (CA19–9) is the most sensitive diagnostic marker for PC, but it is not useful for diagnosing early PC [2]. Therefore, new biomarkers that can detect the initial stages of PC are urgently needed.

Intraductal papillary mucinous neoplasm (IPMN) is a pre-cancerous lesion, and 1–2% of IPMN cases progress to PC each year [3]. IPMN progress from a non-invasive to an invasive lesion [4], and the postoperative prognosis of patients with invasive IPMN appears to be considerably worse than that of patients with non-invasive IPMN [5]. This evidence suggests that biological markers able to distinguish invasive IPMN from non-invasive IPMN can improve the survival of PC patients. While the utility of CA19–9 and MUC5AC as serum markers of malignant IPMN has been reported, their sensitivities were not high enough to be indicative factors for resection [6]. Indeed, even when novel imaging procedures are utilized, it is difficult to predict the malignant potential of IPMN [4]. Novel indicators that can predict the malignant potential of IPMN are therefore eagerly awaited.

MicroRNAs (miRs), which are small RNAs that regulate approximately 30% of human genes [7], are secreted into the blood and body fluids [8]. Recent studies have shown that the abnormal expression of extracellular circulating miRs (CmiRs) in serum or plasma was correlated with the prognosis of PC, suggesting that CmiRs may be potential diagnostic or prognostic markers for advanced PC [9].

miR-21 was reported that proportionally increased during the progression from IPMN to PC, but no other miRs have been identified as markers for the detection of IPMN as well as the progression of PC.

miRs have been reported to be stably contained within vesicles called exosomes [10]. Exosomes are small (40–100 nm diameter) vesicles composed of a lipid bilayer and secreted by cells to interact with distant tissues; they may be found in all body fluids, including the serum and plasma [10–12]. miRs and mRNAs were found to be enclosed in exosomes, stabilized from RNase and highly enriched compared to the serum [11, 12], and the expressions of these exosomal microRNAs (ExmiRs) were dysregulated in several types of cancer patients [10]. ExmiRs are therefore expected to be useful as non-invasive diagnostic biomarkers in cancer patients.

We herein assessed for the first time the expression of ExmiRs in patients with IPMN and PC using a next-generation sequencing analysis, and revealed that three ExmiRs were upregulated in IPMN and PC. In addition, the expression of these ExmiRs was correlated with poor prognosis in PC patients and the high-risk cases in the IPMN group, respectively.

## Methods

### Patients

Thirty-two patients with newly diagnosed PC and 29 with IPMN (no prior treatment) at Asahikawa Medical University Hospital from April 2013 to December 2015 were respectively enrolled in the PC group and IPMN group in this study. Twenty-two patients without malignant or neoplastic lesions were registered in the control group; these patients were recruited from patients who visited the Division of Gastroenterology and Hematology/Oncology in Asahikawa Medical University during the study period. The characteristics of the patients in the control group are shown in Table 1. Six cases complaining of abdominal pain and 1 case complaining of nausea were included. Patients with other cancers or neoplasms were excluded from this study. All patients with PC and IPMN underwent enhanced computed tomography from the chest to the abdominal region for tumor staging, according to either the TNM criteria or the IPMN guidelines. Informed consent was obtained from all of the participants regarding the use of their blood samples in this study. The study was approved by the Medical Ethics Committee of Asahikawa Medical University.

**Table 1:** Characteristics of the control, IPMN, and PC groups

Characteristic		Control	IPMN	PC
Total, n		22	29	32
Sex, n (%)	Female	8 (36.4)	16 (55.2)	15 (46.9)
	Male	14 (63.6)	13 (44.8)	17 (53.1)
Age (mean ± SD)		57.5 ± 15.3	73.8 ± 7.8	64.0 ± 10.1
Stage (UICC) I / Ila / llb / III / IV		–	–	2 / 7 / 4 / 5 / 14
Fukuoka criteria FN / WF / HRS		–	14 / 11 / 4	–
Clinical information		GBP 4		
		Chronic gastritis 3		
		Gallbladder stone 2		
		ADM 2		
		Liver cyst 1		
		IBS 1		
		Accessory spleen 1		
		Only symptom 7		

*SD* standard deviation, *IPMN* intraductal papillary mucinous neoplasm, *PC* pancreatic cancer, *FN* Fukuoka negative, *WF* worry-some Feature, *HRS* high-risk stigmata, *GBP* gallbladder cholesterol polyp, *ADM* adenomyomatosis, *IBS* irritable bowel syndrome

### Serum samples

A blood examination and sampling were performed before treatment, which included surgery, chemotherapy, and radiotherapy. The peripheral blood from patients was collected and then centrifuged at 5000 rpm (rpm) for 10 min at 4 °C. The serums were then transferred to fresh tubes and stored at − 80 °C. Before analysis, the serum samples were filtrated through a 0.45-μm pore membrane (Millipore, Billerica, MA, USA). The amount of serum used in all of this study was unified in 250 μl according to the Manufacture.

### Isolation of the exosomes from the serum and MicroRNA isolation from the exosomes

Exosomes were collected from the serum using ExoQuick Exosome Precipitation Solution (System Biosciences, Mountain View, CA, USA) in accordance with the manufacturer’s instructions. Exosomal RNAs were isolated by using Trizol (Invitrogen, Grand Island, NY, USA) and purified using a mirVana miRNA isolation kit (Life Technologies, Carlsbad, CA, USA). The purity and concentration of all RNA samples were quantified spectrophotometrically using the NanoDrop ND-1000 system (NanoDrop, Wilmington, DE, USA). Exosomes were quantified using a CD63 ExoELISA kit (System Biosciences) in accordance with the manufacturer’s instructions.

### Selection of MicroRNA in the exosome using a next-generation sequencer

Five patients were randomly selected from each groups to examine the expression of their exosomal miR. The volumes of the RNA samples (collected from 250-μl serum samples) was normalized. RNA libraries were generated using an Ion Total RNA-Seq Kit v2 (Life Technologies) in accordance with the manufacturer’s instructions. The RNA libraries were then processed for the emulsion PCR using an Ion OneTouchTM system and an Ion OneTouch 200 Template kit v2 (Life Technologies). Template-positive Ion SphereTM particles were enriched and purified for the sequencing reaction with an Ion OneTouchTM ES system (Life Technologies). The template-positive Ion SphereTM Particles were then applied to Ion PI™ Chips (Life Technologies), and the next-generation sequencing reaction was carried out using an Ion Proton™ Semiconductor sequencer (Life Technologies). All of the sequencing data were mapped on a miR sequence using the CLC Genomics Workbench software program (CLC Bio, Aarhus, Denmark), and an expression analysis was performed for each sample.

### MicroRNA detection by quantitative real-time polymerase chain reaction

miRs were reverse-transcribed, and their expressions were determined by quantitative real-time polymerase chain reaction (qRT-PCR) using TaqMan microRNA assay kits in accordance with the manufacturer’s instructions (Applied Biosystems, Foster City, CA, USA). The Ct values were used in the analysis of the qRT-PCR data.

### Statistical analysis

The expression of miR and CD63 in serum samples was compared using the Mann-Whitney U test (for two groups) or the Kruskal-Wallis test followed by Dunn’s test (for three groups). There was no adjustment for multiple comparisons in the subgroup or multiple miRs analysis. The diagnostic performance was confirmed by Receiver Operating Characteristic (ROC) curve analysis. The cutoff point was determined by the following formula: Distance = (1-sensitivity)^2^ + (1-specificity)^2^.

In survival analyses, the probability of overall survival (OS) was determined by the Kaplan-Meier method with a log-rank test and Cox’s proportional-hazards regression model. The statistical analysis was performed using the Graph Pad PRISM (Version 5.0a; GraphPad Software, Inc. La Jolla, CA, USA), SPSS and R software programs. The level of significance was set at *p* < 0.05.

## Results

### Characteristics of the control, IPMN and PC groups

The subjects comprised 32 patients with PC, 29 patients with IPMN and 22 patients without malignant or neoplastic lesions (Control group). Among the 32 PC patients, 12 underwent surgical resection and 28 received chemotherapy. All of the patients in the IPMN group were diagnosed with branched-duct type (BD)-IPMN. Among the 29 IPMN patients, 15 with Fukuoka Negative (FN) and 11 with Worrisome Features (WF) were conservatively observed, and four cases with High-risk Stigmata (HRS) underwent surgical resection. The conditions of the patients in the Control group (*n* = 22) included gallbladder cholesterol polyp (*n* = 4), chronic gastritis (*n* = 3), gallbladder stone or adenomyomatosis (n = 2), and liver cyst or irritable bowel syndrome or accessory spleen (*n* = 1), the remaining seven only had symptoms and were not diagnosed with any disease. The median age in the IPMN group (73.8 ± 7.8 years) was older than that in the PC (64.0 ± 10.1 years) and control groups (57.5 ± 15.3 years), but no significant difference were noted in gender among the groups (Table 1).

### Serum exosomes were not markedly different between the control, IPMN and PC groups

First, we assessed the concentration of serum exosomes in each group. The control (*N* = 20), IPMN (*N* = 29) and PC groups (*N* = 31) were subjected to this assay. Exosomes were isolated from 250 μL serum using ExoQuick solution and the yields were measured by a CD63, a component of the exosome layer, ExoELISA kit (System Biosciences). No significant differences were noted in the optical density (OD) of CD63 among the groups, indicating no marked differences in the concentration of serum exosomes (Additional file 1: Figure S1).

### ExmiR-191, − 21, and -451a, significantly up-regulated in PC and IPMN, were sensitive diagnostic markers

**Fig. 1:**
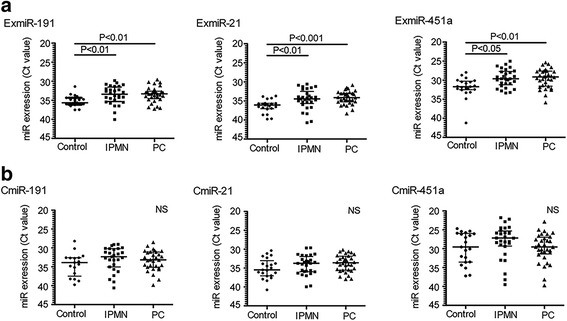
ExmiR-191, − 21 and -451a were significantly up-regulated in PC and IPMN. The three candidate miRs extracted with next-generation sequencing analysis were further evaluated using a qRT-PCR targeting all cases. **a**, **b** The expressions of ExmiR-191 (**a**, left panel), ExmiR-21 (**a**, middle panel), ExmiR-451a (**a**, right panel), CmiR-191 (**b**, left panel), CmiR-21 (**b**, middle panel) and CmiR-451a (**b**, right panel) were plotted (median with interquartile range was also shown). The expression of ExmiR-191, ExmiR-21, and ExmiR-451a were significantly higher in PC (*n* = 32) and IPMN patients (*n* = 29) than in controls (*n* = 22), while the expressions of these CmiRs did not differ significantly among the groups

We analyzed the ExmiR profiles of each group using an exosomal microRNA sequence analysis with next-generation sequencing (*N* = 5 each). Among a total of 347 detected ExmiRs, the expression of ExmiR-191, ExmiR-21 and ExmiR-451a was significantly increased in both the IPMN or PC groups by the Kruskal-Wallis test (Table 2). These three candidates were further evaluated using a qRT-PCR targeting all cases. The expressions of ExmiR-191, − 21 and -451a were significantly higher in PC and IPMN patients than in controls (Fig. 1a). Of note, the expression of CmiR-191, CmiR-21, and CmiR-451a, which are bulky serum miRs including not only ExmiRs but also other serum miRs, did not differ markedly among the groups (Fig. 1b). Since the IPMN patients were significantly older, the age-adjusted differences were evaluated; no significant interaction was found between any of the ExmiRs and age (ExmiR-191, *p* = 0.932; ExmiR-21, *p* = 0.478; ExmiR-451a, *p* = 0.357).

**Table 2:** ExmiR-191, −21 and -451a were identified as candidates for biological markers of IPMN and PC by next-generation sequencing analysis

Candidate ExmiR	*P* value	Fold change (Control vs IPMN)	Fold change (Control vs PC)
ExmiR-191	0.0036	3.171	34.571
ExmiR-21	0.0417	12.222	25.556
ExmiR-451a	0.0477	1.819	11.662

**Fig. 2:**
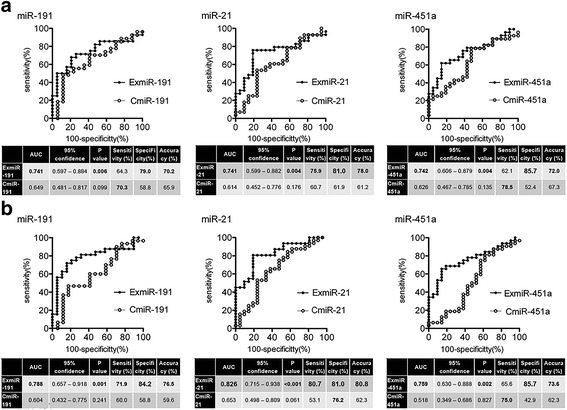
Three ExmiRs were more sensitive markers for diagnosing IPMN and PC than CmiRs. **a**,**b** The ROC analysis between control and IPMN (**a**) or PC (**b**) was showed, as follows: miR-191 (left panel), miR-21 (middle panel), and miR-451a (right panel). The AUC, specificity and diagnostic accuracy of three ExmiRs were superior to those of three CmiRs. Among the three ExmiRs, ExmiR-21 showed the highest diagnostic accuracy (IPMN diagnostic accuracy = 78.0%, PC diagnostic accuracy = 80.8%)

To evaluate the diagnostic performance of three ExmiRs, ROC curve analysis was performed. The ROC analysis between control and IPMN (Fig. 2a) or PC (Fig. 2b) showed that the area under the curve (AUC), diagnostic accuracy and specificity of the three ExmiRs were superior to those of the three CmiRs. The accuracy of the ExmiRs was almost 5–20% higher than that of the CmiRs. Among the three ExmiRs, ExmiR-21 showed the largest AUC and highest diagnostic accuracy (IPMN diagnosis: accuracy = 78.0%, PC diagnosis: accuracy = 80.8%). These results indicated that ExmiRs were more sensitive markers for diagnosing IPMN and PC than CmiRs.

### ExmiR-191, ExmiR-21, and ExmiR-451a were good diagnostic markers for IPMN and early-stage PC

**Fig. 3:**
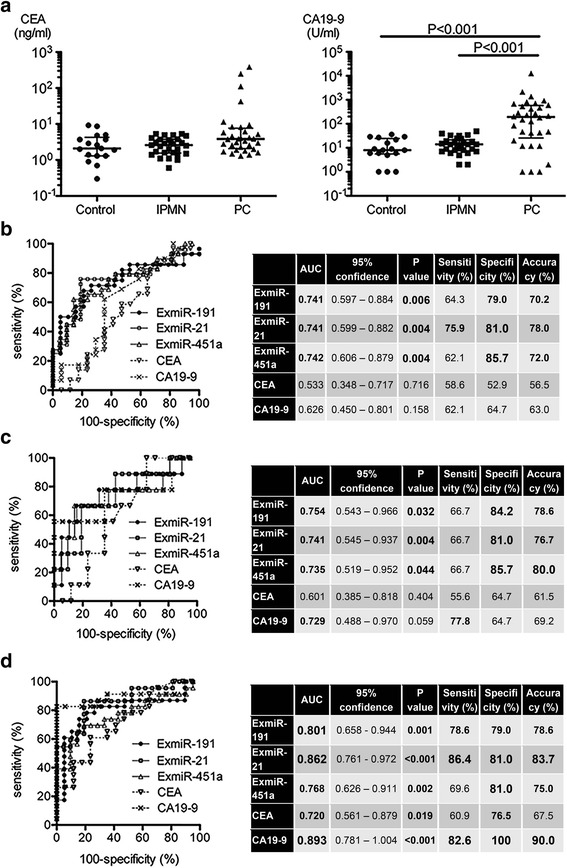
ExmiR-191, ExmiR-21, and ExmiR-451a were good diagnostic markers for IPMN and early-stage PC. **a** The levels of CEA (left) and CA19–9 (right) were plotted (median with interquartile range was also shown). CA19–9 was significantly higher in the PC group than in the control or IPMN groups. **b** The ROC analysis between control and IPMN was shown. The AUC and diagnostic accuracy of three ExmiRs were clearly superior to traditional markers. **c** The ROC analysis between control and early stage of PC including only patients with stage I or IIa showed. The diagnostic accuracy of three ExmiRs were superior to CEA, and tended to be better than CA19–9. **d** The ROC analysis between the control group and the advanced-stage PC group (stage ≥IIb). The AUC values and accuracy of the three ExmiRs were superior to CEA, but they were not as useful as CA19–9

We identified three ExmiRs as biological markers for diagnosing early-stage pancreatic tumorigenesis. We next compared the diagnostic performance of the three ExmiRs with those of carcinoembryonic antigen (CEA) and carbohydrate antigen 19–9 (CA19–9), which are traditional markers for PC. The levels of CA19–9 were significantly higher in the PC group than in the control or IPMN groups (Fig. 3a).

In the ROC analysis between control and IPMN, the AUC and diagnostic accuaracy of ExmiRs were superior to those of CA19–9 and CEA (Fig. 3b). In addition, ROC analysis between control and earlier stages of PCs including patients with stage I and IIa showed that the accuracy of the ExmiRs was preferable to that of CEA. There was no significant difference in comparison to CA19–9; however, the positive detection rate was approximately 10% higher (Fig. 3c). On the other hand, CA19–9 was the best parameter for the diagnosis of advanced-stage PC (stage ≥IIb) (AUC 0.893, accuracy 90%). The ExmiR’s, which were somewhat inferior to CA19–9, showed a good AUC value and accuracy (ExmiR-21, AUC 0.862, accuracy 83.7%) (Fig. 3d). Taken together, our results suggested that ExmiR-191, ExmiR-21 and ExmiR-451a were good diagnostic markers for IPMN and early-stage PC, but that CA19–9 was still superior for the diagnosis of advanced cancer.

### The expression of ExmiR-451a was associated with mural nodules of IPMN.

**Fig. 4:**
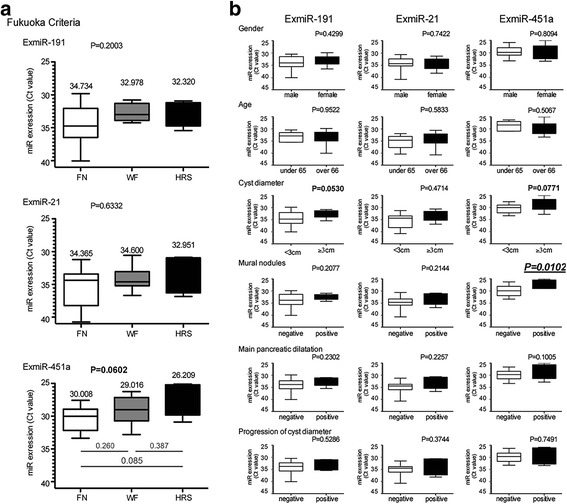
ExmiR-451a was correlated with the Clinical Features of IPMN, and might be able to diagnose high risk cases. **a** The correlation between the expression levels of the three ExmiRs and the Fukuoka criteria. There were no significant differences in the expression of ExmiR-191, ExmiR-21 or ExmiR-451a between FN, WF and HRS. However, the ExmiR-451a level appeared to be gradually increasing (*p* = 0.0602). **b** The association between the expressions of three ExmiRs and clinical features, gender, age, cyst diameter, mural nodules, main pancreatic dilatation and Progression of cyst diameter, in IPMN group was assessed. The ExmiR expressions were plotted and those median with interquartile range was also shown. The expression of ExmiR-451a was significantly higher in the patients with mural nodules. Although there were no other significant differences, ExmiR-451a also seemed to be high with a large cyst diameter and main pancreatic dilatation

The international consensus guideline 2012 for IPMN describes the indication criteria for resection, known as the Fukuoka Criteria. According to the Fukuoka Criteria, IPMNs are categorized as FN, WF and HRS, and these three categories are generally said to reflect progression from benign IPMN to malignant IPMN. The Kruskal-Wallis test revealed no significant differences in the expression levels of ExmiR-191, ExmiR-21 and ExmiR-451a among FN, WF and HRS (Fig. 4a). However, the ExmiR-451a level appeared to gradually increase; we therefore decided to evaluate the associations between ExmiR-451a and each of the factors in the Fukuoka criteria (cyst diameter, presence of mural nodules, presence of main pancreatic duct dilatation, progression of cyst diameter). The expression of ExmiR-451a was significantly higher in the patients with mural nodules than in those without them (Fig. 4b). Although there were no other significant differences, ExmiR-451a also seemed to be high in the IPMN patients with large cyst diameter and main pancreatic dilatation. ExmiR-451a might be strongly associated with the malignant progression of IPMN.

### ExmiR-21 was a candidate prognostic factor for the survival of PC patients

**Fig. 5:**
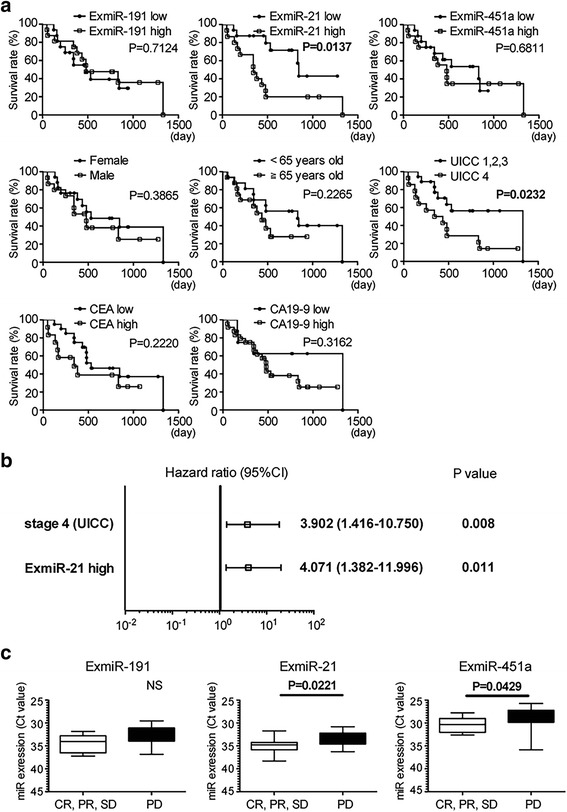
ExmiR-21 was a candidate prognostic factor for the survival of PC patients. The association between the three ExmiRs and the clinical outcomes in PC patients was evaluated. **a**, **b** PC patients were categorized into high- and low-ExmiR expression groups using the median miR value as the cut-off point. **a** The survival analysis by Kaplan-Meier method with a log-rank test was shown. The overall survival in the high-ExmiR-21 expression group was significantly shorter than that in the low-ExmiR-21 expression group (*p* = 0.0137, median OS of low-ExmiR-21 expression group: 846 days, median OS of high-ExmiR-21 expression group: 344 days), but ExmiR-191 and ExmiR-451a were not associated with the survival of PC patients. With regard to the other factors, UICC4 was a significant prognostic factor (*p* = 0.0232, median OS of UICC1,2,3: 1330 days, median OS of UICC4: 388.5 days). **b** Multivariate survival analysis using Cox’s proportional-hazards regression model was performed to assess the relationship between the overall survival and the following candidate prognostic factors that were found to be significant by the Kaplan–Meier method: UICC stage (stage IV) and ExmiR-21. UICC stage IV (hazard ratio: 3.902, 95% CI: 1.416–10.750) and the high expression of ExmiR-21 (hazard ratio: 4.071, 95%CI: 1.382–11.996) were identified as independent prognostic factors. **c** The association between the expressions of three ExmiRs and the clinical outcome of chemotherapy was also assessed. The ExmiR expressions were plotted and those median with interquartile range was also shown. The expression of ExmiR-21 and ExmiR-451a in the group with disease progression (PD) was significantly higher than in the group with complete response (CR), partial response (PR), or stable disease (SD) (*p* = 0.0221, *p* = 0.0429, respectively)

We finally focused on the PC group and evaluated the association between the three ExmiRs and the clinical outcomes in PC patients. For the survival analysis, PC patients were categorized into high- and low-ExmiR expression groups using the median miR value as the cut-off point. Survival curves of the three ExmiRs estimated by the Kaplan-Meier method are shown in Fig. 5a. The overall survival in the high-ExmiR-21 expression group (median: 344 days) was significantly shorter than that in the low-ExmiR-21 expression group (median: 846 days), but ExmiR-191 and ExmiR-451a did not affect the survival of PC patients. With regard to the other factors, UICC4 was a significant prognostic factor (*p* = 0.0232, median OS of UICC1,2,3: 1330 days, median OS of UICC4: 388.5 days). We also performed a multivariate survival analysis using Cox’s proportional-hazards regression model to assess the relationship between the overall survival and the following candidate prognostic factors that were identified as significant by the Kaplan–Meier method (Fig. 5b). Both UICC stage IV (hazard ratio: 3.902, 95% CI: 1.416–10.750) and the high expression of ExmiR-21 (hazard ratio: 4.071, 95%CI: 1.382–11.996) were identified as independent prognostic factors for the overall survival.

In addition, we also evaluated the association between the clinical outcome of chemotherapy and the three ExmiRs. The expression of ExmiR-21 and ExmiR-451a in the group with progression disease (PD) was significantly higher than in the groups with complete response (CR), partial response (PR), or stable disease (SD) (*p* = 0.022, *p* = 0.043, respectively) (Fig. 5c). This result might suggest that ExmiR-21 and ExmiR-451a reduced the disease control rate in PC patients.

## Discussion

The present study analyzed for the first time the serum ExmiRs in PC and IPMN patients using a next-generation sequencing, resulting that ExmiR-191, ExmiR-21, and ExmiR-451a were identified as candidate miRs which are dysregulated in IPMN and PC patients. The qRT-PCR confirmed that the expressions of ExmiR-191, ExmiR-21, and ExmiR-451a were increased in the patients with PC and IPMN.

Previous reports have suggested that CmiRs are useful for detecting or determining the prognosis of PC, invasive IPMNs, and other cancers [12, 13]. In the present study, we showed that the expressions of ExmiR-191, ExmiR-21 and ExmiR-451a were significantly up-regulated in PC and IPMN. However, of note: the expressions of CmiR-191, CmiR-21, and CmiR-451a were not markedly changed between the control, IPMN, and PC groups, illustrating the utility of ExmiRs as detection makers of PC and IPMN over CmiRs. CmiRs have been reported to be stabilized in vesicles such as exosomes [14], and the exosomes in serum are highly enriched in miRs [11]. Tanaka et al. also showed that circulating miR-21 originated from exosomes, as the miR-21 expression was significantly higher in exosomes than in the serum remaining after exosome extraction [12]. These present and previous findings therefore suggest that ExmiRs are more useful as markers for tumor detection than CmiRs.

It should be noted that the current established tumor markers were elevated in the advanced cancers, but not in IPMN, while the ExmiRs were upregulated in both IPMN and PC, including both early and advanced phases. The diagnostic performance estimated by the ROC curve analysis favorable AUC and accuracy as compared to CEA and CA19–9 in IPMN and early stage of PC, suggesting the levels of ExmiR-191, ExmiR-21, and ExmiR-451a can thus serve as early diagnostic markers of pancreatic neoplasms.

miR-191 has been reported to be up-regulated in a wide range of human cancers, including PC [15]. miR-191 might be responsible for the abnormal expression of many target genes such as CDK9, NOTCH2, and RPS6KA3 [16], as these genes have been reported to be direct targets of miR-191 and regulators of proliferation. In addition, miR-191 was found to regulate cell invasion and differentiation, facilitate extracellular matrix formation, and encourage metastasis [15]. miR-191 was also found to up-regulate p53 deletion and inhibit the expression of the tumor-suppressive mRNA, C/EBPβ, thereby enhancing the tumor progression in colorectal cancer [17]. Taken together, the findings from these previous studies show the oncogenic features of miR-191, which supports our finding that ExmiR-191 is a candidate diagnostic marker of pancreatic neoplasms.

miR-21 is also considered an oncogenic miR because it is up-regulated in various cancers and targets the tumor-suppressive mRNAs [18]. Previous reports of basic studies have stated that overexpression of miR-21 promoted cellular proliferation, survival, and invasion and migration of cancer cells, including PC cells [19, 20]. The overexpression of miR-21 down-regulated the expression of tumor suppressors such as PDCD4 and TIMP3 and promoted cell proliferation, leading to the development of PC [21, 22]. Conversely, the suppression of miR-21 reduced cancer cell survival and tumor growth in a murine xenograft model [23]. Indeed, clinical studies have shown that miR-21 was up-regulated in invasive IPMN compared with noninvasive IPMN [24] and was up-regulated in noninvasive IPMN compared to normal pancreatic tissue. In the present study, ExmiR-21 was identified as an early diagnostic marker of pancreatic neoplasms, a finding which is consistent with those of previous studies. Recent studies have indeed reported that miR-21 reduced the sensitivity of cancer cells to anticancer drugs such as gemcitabine and 5-FU-based chemotherapy [25], and the suppression of miR-21 increased sensitivity to gemicitabine and induced apoptosis in PC patients [26]. Furthermore, the overexpression of miR-21 correlates with a poor prognosis after PC resection, independent of other clinicopathologic factors [27]. Interestingly, ExmiR-21 was also identified as a chemo-sensitive marker and a prognostic factor for the overall survival in this study. Taken together, these present and previous findings suggest that miR-21 plays a role in pancreatic carcinogenesis.

miR-451a is located on chromosome 17q11.2 in humans and has been reported to suppress cell proliferation and colony formation by targeting the Ywhaz (14–3-3zeta) gene, RAB14 protein, and activating transcription factor 2 (ATF2). It is therefore considered to be a tumor-suppressive miR in several human malignancies [28, 29]. In addition, a clinical study showed that miR-451a expression was down-regulated in cancer tissue, and low miR-451a expression tends to be associated with metastasis and shorter survival duration [30]. These findings suggest that miR-451a acts as a tumor suppressor. In our analysis, ExmiR-451a expression was significantly increased in both the IPMN and PC groups. A high expression of ExmiR-451a might be induced by a positive feedback system due to the progression of the pancreatic tumor. Further analyses of the miR-451a expression in IPMN and PC cells will be needed to fully clarify the role of miR-451a in the development of PC.

The present study was associated with some limitations. We investigated biomarkers using serum samples from 32 patients with PC, 29 patients with IPMN and 22 healthy volunteers as a discovery cohort. To validate the efficacy of three ExmiRs, further studies should be conducted using large cohorts. In addition, the present study focused on IPMN and PC, and did not include cases with other pancreatic cystic neoplasms. A further analysis that includes patients with cystic neoplasms as well as those with non-neoplasms should be performed to identify biomarkers that can be used to distinguish IPMN from other cystic lesions.

## Conclusions

In summary, the present study revealed that serum levels of ExmiR-191, ExmiR-21, and ExmiR-451a are up-regulated in PC and IPMN patients. These findings encourage the development of a novel non-invasive strategy for diagnosing pancreatic neoplasm by determining the expressions of ExmiR-191, − 21 and -451a enclosed in exosomes.

## Additional file

Additional file 1: Table S1. The differences in the expression of exosomes between the IPMN and PC patients. An ELISA revealed no significant difference in the CD63 expression of exosomes in the serum samples, indicating that the expression of exosomes did not markedly differ among the groups (PC = 31, IPMN = 29, control = 20).

## Abbreviations

ATF2: activating transcription factor 2
AUC: Area under the curve
CmiR: Circulating microRNA
CR: Complete response
ExmiR: Exosomal microRNA
FN: Fukuoka negative
HRS: High-risk stigmata
IPMN: Intraductal papillary mucinous neoplasm
miR: microRNA
OD: Optical density
OS: Overall survival
PC: Pancreatic cancer
PD: Progression disease
PR: Partial response
ROC: Receiver operating characteristic
rpm: Revolutions per minute
SD: Stable disease
WF: Worrisome feature

## References

[1] Siegel RL, Miller KD, Jemal A (2015). Cancer statistics, 2015. CA Cancer J Clin.

[2] Ballehaninna UK, Chamberlain RS (2012). The clinical utility of serum CA 19-9 in the diagnosis, prognosis and management of pancreatic adenocarcinoma: an evidence based appraisal. Journal Of Gastrointestinal Oncology.

[3] Uehara H, Nakaizumi A, Ishikawa O, Iishi H, Tatsumi K, Takakura R (2008). Development of ductal carcinoma of the pancreas during follow-up of branch duct intraductal papillary mucinous neoplasm of the pancreas. Gut.

[4] Maguchi H, Tanno S, Mizuno N, Hanada K, Kobayashi G, Hatori T (2011). Natural history of branch duct intraductal papillary mucinous neoplasms of the pancreas: a multicenter study in Japan. Pancreas.

[5] Nagai K, Doi R, Kida A, Kami K, Kawaguchi Y, Ito T (2008). Intraductal papillary mucinous neoplasms of the pancreas: clinicopathologic characteristics and long-term follow-up after resection. World J Surg.

[6] Tanaka M, Castillo C F-d, Adsay V, Chari S, Falconi M (2012). International consensus guidelines 2012 for the management of IPMN and MCN of the pancreas. Pancreatology.

[7] Lim LP, Lau NC, Garrett-Engele P, Grimson A, Schelter JM, Castle J (2005). Microarray analysis shows that some microRNAs downregulate large numbers of target mRNAs. Nature.

[8] Calin GA, Croce CM (2006). MicroRNA signatures in human cancers. Nat Rev Cancer.

[9] Le Large TY, Meijer LL, Mato Prado M, Kazemier G, Frampton AE, Giovannetti E (2015). Circulating microRNAs as diagnostic biomarkers for pancreatic cancer. Expert Rev Mol Diagn.

[10] Zöller M (2013). Pancreatic cancer diagnosis by free and exosomal miRNA. World J Gastrointest Pathophysiol.

[11] Gallo A, Tandon M, Alevizos I, Illei GG (2012). The majority of microRNAs detectable in serum and saliva is concentrated in exosomes. PLoS One.

[12] Tanaka Y, Kamohara H, Kinoshita K, Kurashige J, Ishi T, Iwatsuki M (2013). Clinical impact of serum exosomal microRNA-21 as a clinical biomarker in human esophageal squamous cell carcinoma. Cancer.

[13] Giovannetti E, Funel N, Peters GJ, Del Chiaro M, Erozenci LA, Vasile E (2010). MicroRNA-21 in pancreatic cancer: correlation with clinical outcome and pharmacologic aspects underlying its role in the modulation of gemcitabine activity. Cancer Res.

[14] Köberle V, Pleli T, Schmithals C, Augusto Alonso E, Haupenthal J, Bonig H (2013). Differential stability of cell-free circulating microRNAs: implications for their utilization as biomarkers. PLoS One.

[15] Song Z, Ren H, Gao S, Zhao X, Zhang H, Hao J (2014). The clinical significance and regulation mechanism of hypoxia-inducible factor-1 and miR-191 expression in pancreatic cancer. Tumor Biol.

[16] Polioudakis D, Abell NS, Iyer VR (2015). MiR-191 regulates primary human fibroblast proliferation and directly targets multiple oncogenes. PLoS One.

[17] Zhang XF, Li KK, Gao L, li SZ, Chen K, Zhang JB (2015). miR-191 promotes tumorigenesis of human colorectal cancer through targeting C/EBPβ. Oncotarget.

[18] Pan X, Wang ZX, Wang R (2010). MicroRNA-21: a novel therapeutic target in human cancer. Cancer Biol Ther.

[19] Moriyama T, Ohuchida K, Mizumoto K, Yu J, Sato N, Nabae T (2009). MicroRNA-21 modulates biological functions of pancreatic cancer cells including theirproliferation, invasion, and chemoresistance. Mol Cancer Ther.

[20] Kadera BE, Li L, Toste PA, Wu N, Adams C, Dawson DW (2013). MicroRNA-21 in pancreatic ductal adenocarcinoma tumor-associated fibroblasts promotes metastasis. PLoS One.

[21] Hiyoshi Y, Kamohara H, Karashima R, Sato N, Imamura Y, Nagai Y (2009). MicroRNA-21 regulates the proliferation and invasion in esophageal squamous cell carcinoma. Clin Cancer Res.

[22] Nagao Y, Hisaoka M, Matsuyama A, Kanemitsu S, Hamada T, Fukuyama T (2012). Association of microRNA-21 expression with its targets, PDCD4 and TIMP3, in pancreatic ductal adenocarcinoma. Mod Pathol.

[23] Frezzetti D, De Menna M, Zoppoli P, Guerra C, Ferraro A, Bello AM (2011). Upregulation of miR-21 by Ras in vivo and its role in tumor growth. Oncogene.

[24] Matthei H, Wylie D, Lloyd M, Dal Molin M, Kemppainen J, Mayo SC (2012). MicroRNA biomarkers in cyst fluid augment the diagnosis and management of pancreatic cysts. Clin Can Res.

[25] Hong L, Han Y, Zhang Y, Zhang H, Zhao Q, Wu K (2013). MicroRNA-21: a therapeutic target for reversing drug resistance in cancer. Expert Opin Ther Targets.

[26] Park JK, Lee EJ, Esau C, Schmittgen TD (2009). Antisense inhibition of microRNA-21 or −221 arrests cell cycle, induces apoptosis, and sensitizes the effects of gemcitabine in pancreatic adenocarcinoma. Pancreas.

[27] Frampton AE, Krell J, Jamieson NB, Gall TM, Giovannetti E, Funel N (2015). microRNAs with prognostic significance in pancreatic ductal adenocarcinoma: a meta-analysis. Eur J Cancer.

[28] Wang R, Wang ZX, Yang JS, Pan X, De W, Chen LB (2011). MicroRNA-451 functions as a tumor suppressor in human non-small cell lung cancer by targeting ras-related protein 14 (RAB14). Oncogene.

[29] Li Y, Wang J, Dai X, Zhou Z, Liu J, Zhang Y (2015). miR-451 regulates FoxO3 nuclear accumulation through Ywhaz in human colorectal cancer. Am J Transl Res.

[30] Su Z, Zhao J, Rong Z, Geng W, Wang Z (2015). MiR-451, a potential prognostic biomarker and tumor suppressor for gastric cancer. Int J Clin Exp Pathol.

