# Supplementary figures and images for: An elevated expression of serum exosomal microRNA-191, − 21, −451a of pancreatic neoplasm is considered to be efficient diagnostic marker
Source: BMC Cancer. 2018 Jan 31;18:116. doi: 10.1186/s12885-018-4006-5 (PMC5793347; doi:10.1186/s12885-018-4006-5)

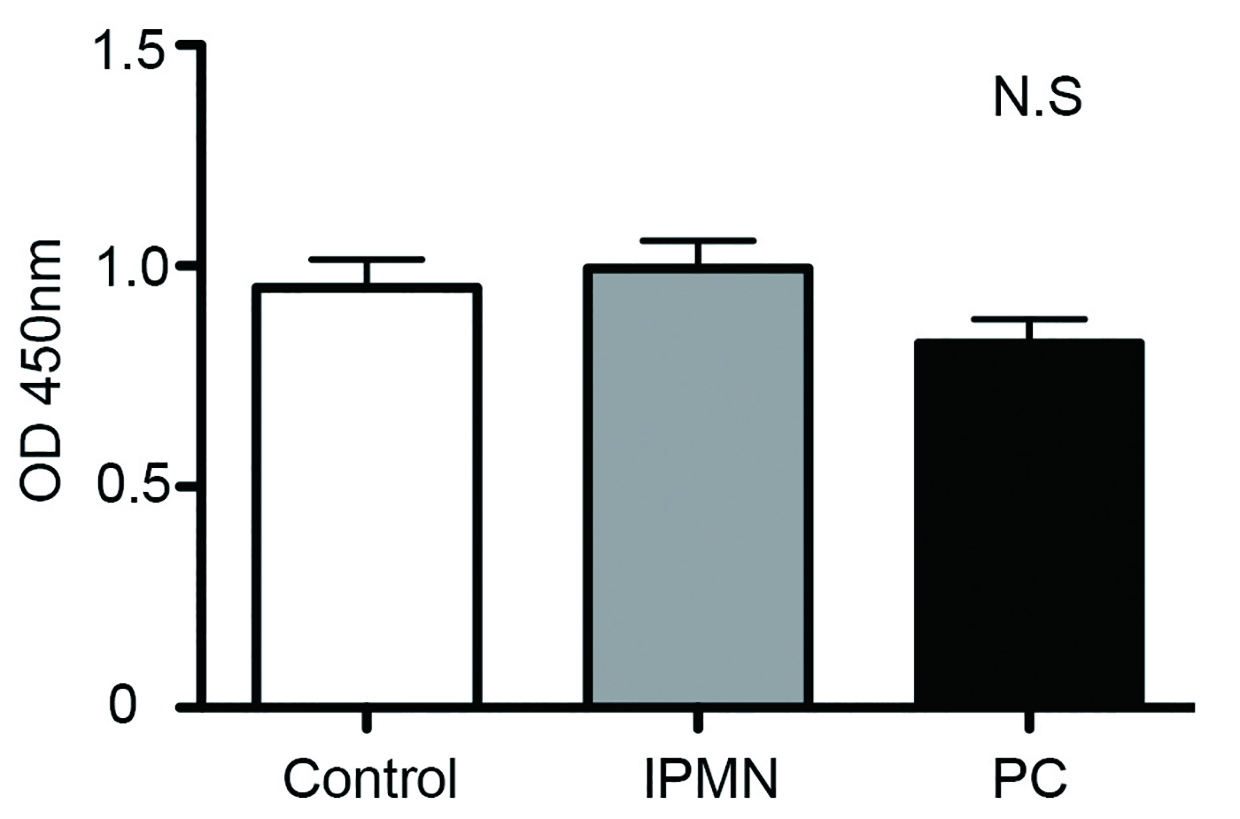

Supplement: Additional file 1: Table S1. — The differences in the expression of exosomes between the IPMN and PC patients. An ELISA revealed no significant difference in the CD63 expression of exosomes in the serum samples, indicating that the expression of exosomes did not markedly differ among the groups (PC = 31, IPMN = 29, control = 20). [file 12885_2018_4006_MOESM1_ESM.tif]
